# Neuroprotective effects of hypoactive *Akkermansia muciniphila* in MPTP-induced mouse models of Parkinson’s disease

**DOI:** 10.1128/spectrum.03379-24

**Published:** 2025-11-12

**Authors:** Jiahui Xi, Chengcheng Tang, Xian Xiao, Min Chen, Qingjian Zou, Xiaoqing Zhou

**Affiliations:** 1 Guangdong Provincial Key Laboratory of Large Animal Models for Biomedicine, South China Institute of Large Animal Models for Biomedicine, School of Pharmacy and Food Engineering, Wuyi University, Jiangmen, China; 2 Sanya Institute, Hainan Academy of Agricultural Sciences, Sanya, China; Uniwersytet Medyczny w Bialymstoku, Bialystok, Poland

**Keywords:** hypoactive *Akkermansia muciniphila*, Parkinson’s disease, neuroinflammation

## Abstract

Parkinson’s disease (PD) has been associated with the microbiota-gut-brain axis, and probiotics have been shown to alleviate PD symptoms via various mechanisms. *Akkermansia muciniphila* has demonstrated therapeutic effects in some neurological disorders; however, its role in PD treatment remains debated. This study revealed that orally administered hypoactive *Akkermansia muciniphila* inhibited dopaminergic neuron loss in a 1-methyl-4-phenyl-1,2,3,6-tetrahydropyridine (MPTP)-induced PD mouse model. Its ingestion also reduced astrocyte activation and mitigated inflammatory responses in both the brain and colon. Additionally, hypoactive *Akkermansia muciniphila* modulated intestinal microbiota composition in PD model mice. These findings suggest that hypoactive *Akkermansia muciniphila* reduces neurotoxicity in an MPTP-induced PD model and may offer potential for inhibiting PD progression.

**IMPORTANCE:**

Recent research suggests a connection between Parkinson’s disease (PD) and the microbiota-gut-brain axis, with evidence indicating that probiotics may alleviate PD symptoms. Although *Akkermansia muciniphila* has demonstrated potential benefits for certain neurological disorders, its efficacy in the treatment of PD is still a subject of ongoing debate. Here, we demonstrated that hypoactive *Akkermansia muciniphila* ameliorated dopaminergic neuronal death, correlating to the reduction of glial hyperactivation and neuroinflammation. Hypoactive *Akkermansia muciniphila* also induced microbiota fluctuation, which may perform a sophisticated effect on PD progression. Our study may provide an innovative strategy for using hypoactive *Akkermansia muciniphila* as a therapy or adjuvant therapy for PD.

## INTRODUCTION

Parkinson’s disease (PD) is the second most common neurodegenerative disorder, with an estimated global incidence of approximately one million cases in 2019 (1). This figure is expected to rise significantly in the coming decades according to recent studies (2). The primary clinical manifestations of PD include motor symptoms, such as bradykinesia, resting tremor, and rigidity, along with alterations in posture and gait (3). Furthermore, patients with PD often experience a variety of non-motor symptoms, which may include hyposmia, constipation, urinary dysfunction, memory loss, depression, and pain (3). Current research indicates that PD is associated with gastrointestinal dysfunction (4), autonomic dysfunction (5), and psychosis (6). The prevailing theory regarding the pathogenesis of PD suggests that it primarily stems from nigrostriatal insufficiency due to the degeneration of dopaminergic neurons in the midbrain substantia nigra (7, 8). Additionally, Lewy bodies (LBs) represent a critical pathological hallmark of the disease, composed of various proteins, including α-synuclein, parkin, ubiquitin, and neurofilaments (9). Notably, some studies have shown that LBs may be present in the gastrointestinal tract before they are detected in the central nervous system (CNS) (10–12), potentially linking them to gastrointestinal symptoms such as constipation commonly observed in PD patients (13).

The bidirectional communication between the gut and brain, which involves immune, endocrine, and metabolic pathways, has been substantiated by an extensive body of research. The microorganisms in the gut play a key regulatory role in this complex communication system and have been extensively studied in various neurological disorders (14–16). Gut microbes may influence neural development and modulate neurotransmission (15). Conversely, the CNS can directly affect gut microbiota through stress mediator-induced virulence gene expression (16). This interconnection is encapsulated by the microbiome-gut-brain axis (MGBA). Numerous studies have demonstrated that the gut and microbiome are closely associated with the progression of PD. Researchers have identified significant differences in gut microbiome composition between PD patients and healthy people (17), and alterations in short-chain fatty acids (SCFAs) produced by the microbiome may contribute to the etiology of PD (17). Additionally, PD patients have been found to exhibit significantly greater intestinal permeability compared to healthy individuals, along with an increase in intestinal α-synuclein (18). Furthermore, fecal microbiota transplantation (FMT) can correct gut microbiota dysbiosis and ameliorate symptoms in a rotenone-induced PD mouse model (19). In a clinical trial, FMT improved gastrointestinal disorders and led to a marked increase in the complexity of the microecological system in patients (20).

Probiotics, defined as live micro-organisms with established beneficial effects on human health, have been studied in the context of various diseases and are utilized as adjuvant treatments (21). It has been demonstrated that probiotics possess supportive therapeutic efficacy in multiple neuropsychiatric disorders, including Alzheimer’s disease (22), multiple sclerosis (23), and depressive disorder (24). In studies of PD, probiotic therapy has been investigated in PD model mice and has been shown to improve symptoms (25). *Akkermansia muciniphila,* a notable probiotic, has been recognized for its diverse beneficial effects on the host and has been studied in various neurological and psychiatric disorders (26–28), suggesting potential therapeutic effects (29).

However, the role of *Akkermansia muciniphila* in PD remains controversial. Some researchers have found that *Akkermansia muciniphila* exerts beneficial effects in a PD mouse model by alleviating neuroinflammation and promoting neurogenesis (30). Conversely, excessive enrichment of *Akkermansia muciniphila* in specific intestinal microenvironments may exacerbate inflammation and damage the epithelial barrier (31). Interestingly, *Akkermansia muciniphila* has also been shown to induce mitochondrial calcium overload and α-synuclein aggregation in an enteroendocrine cell line (32).

The study demonstrates that the purified membrane protein (Amuc_1100) derived from *Akkermansia muciniphila* or pasteurized bacterial preparations can enhance metabolic function in obese mice (33) and attenuate colitis-associated tumorigenesis through the modulation of CD8^+^ T cells in murine models (34). Building upon these findings, we aimed to investigate whether hypoactive *Akkermansia muciniphila* could provide additional therapeutic benefits in a model of PD, while reducing the potential adverse effects related to the administration of live bacteria. We evaluated the neuroprotective properties of hypoactive *Akkermansia muciniphila* in a 1-methyl-4-phenyl-1,2,3,6-tetrahydropyridine (MPTP)-induced PD mouse model, focusing on neuroprotective and inflammatory factors associated with the disease. To further investigate the therapeutic potential of hypoactive *Akkermansia muciniphila* as an adjunctive therapy for Parkinson’s disease, we established an experimental cohort receiving combined treatment with AKK and levodopa, the first-line pharmacological intervention for PD, with a levodopa monotherapy group serving as the positive control. Additionally, we conducted further analyses of fecal SCFA concentrations and gut microbiota compositions to investigate the potential neuroprotective effects of hypoactive *Akkermansia muciniphila*, particularly regarding its influence on the MGBA. Our results indicate that hypoactive *Akkermansia muciniphila* maintains anti-inflammatory effects in PD mice, although its neuroprotective efficacy is limited.

## RESULTS

### The therapeutic effects of hypoactive *Akkermansia muciniphila* on MPTP-induced motor impairments

**Fig 1:**
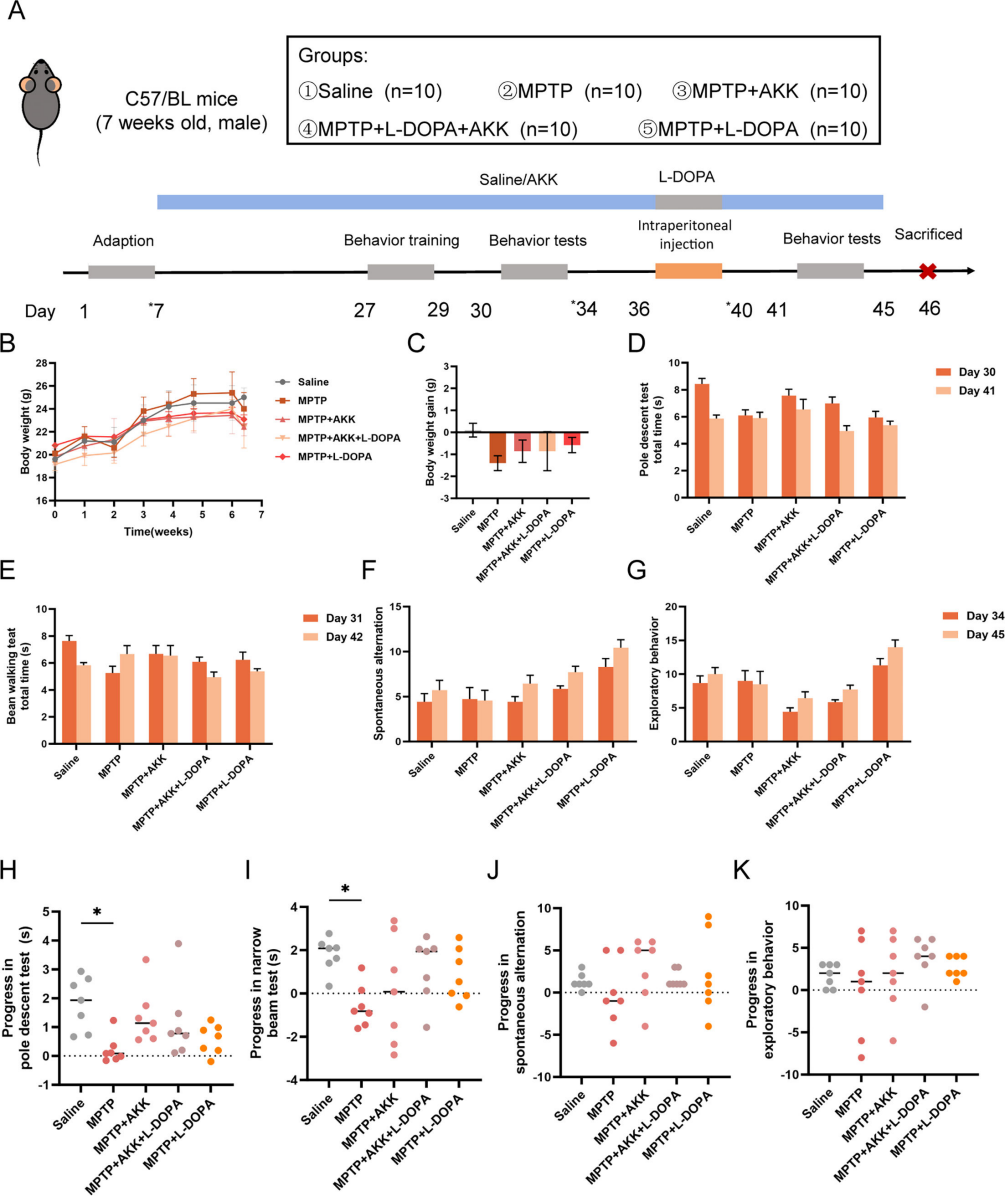
Investigation of the impact of hypoactive *Akkermansia muciniphila* on MPTP-induced behavioral alterations in mice. (**A**) Timeline of the experimental procedure; (**B**) longitudinal analysis of mouse body weight; (**C**) body weight gain following MPTP administration; (**D**) total descent duration in the pole test (*n* = 7); (**E**) total walking duration in the beam walking test (*n* = 7); (**F**) exploratory behavior in the Y-maze (*n* = 7); (**G**) spontaneous alternation in the Y-maze (*n* = 7). Comparative analysis of pre- and post-MPTP treatment performance in the pole test (**H**), beam walking test (**I**), and Y-maze (**J and K**). Data are presented as means ± SEM and were subjected to one-way ANOVA followed by Tukey’s post hoc test. *P < 0.05, indicating a significant difference compared to the saline group.

The experimental design is illustrated in Fig. 1A. All mice in each group exhibited a gradual weight gain prior to the development of the PD model (day 36), with slight fluctuations in this upward trend likely due to individual differences (Fig. 1B). Following the administration of MPTP, the mice began to lose weight, and the resulting weight changes are depicted in Fig. 1C. Notably, the MPTP group experienced significant weight loss compared to the saline group, whereas AKK and therapy slightly reduced the weight loss observed among three therapy groups. Motor function assessments included the pole test (PT) and the narrow-beam test (NBT), both of which were utilized to evaluate the locomotor coordination of the PD mice. The completion times for each test, measured both before and after injection, are presented in Fig. 1D and E. The results indicate that the mice required less time to complete the tests following injection, which may be attributed to the increased proficiency the mice developed through repeated testing. To provide a more direct comparison of differences between groups, we measured the reduction in time in the second test compared to the first test (Fig. 1H and I). Mice in the MPTP group displayed a lesser reduction in time compared to the saline group, indicating motor impairments. Although hypoactive *Akkermansia muciniphila* therapy appeared to offer mild motor-promoting effects, these results were not statistically significant. Neither combined treatment nor monotherapy demonstrated significant therapeutic efficacy. Additionally, we conducted the Y-maze test to assess the spatial exploration capabilities of the mice (Fig. 1F, G, J, and K). MPTP treatment did not result in notable impacts in this experiment, with only minimal significant differences observed between the groups. The data suggest that whereas hypoactive *Akkermansia muciniphila* therapy may not provide significant improvements in motor function, it does not exacerbate the spatial exploration deficits typically seen in PD mice. Further research is required to fully understand the implications of these findings and to explore potential modifications to the therapy.

### Hypoactive *Akkermansia muciniphila* alleviates MPTP-induced dopaminergic neuronal impairments

**Fig 2:**
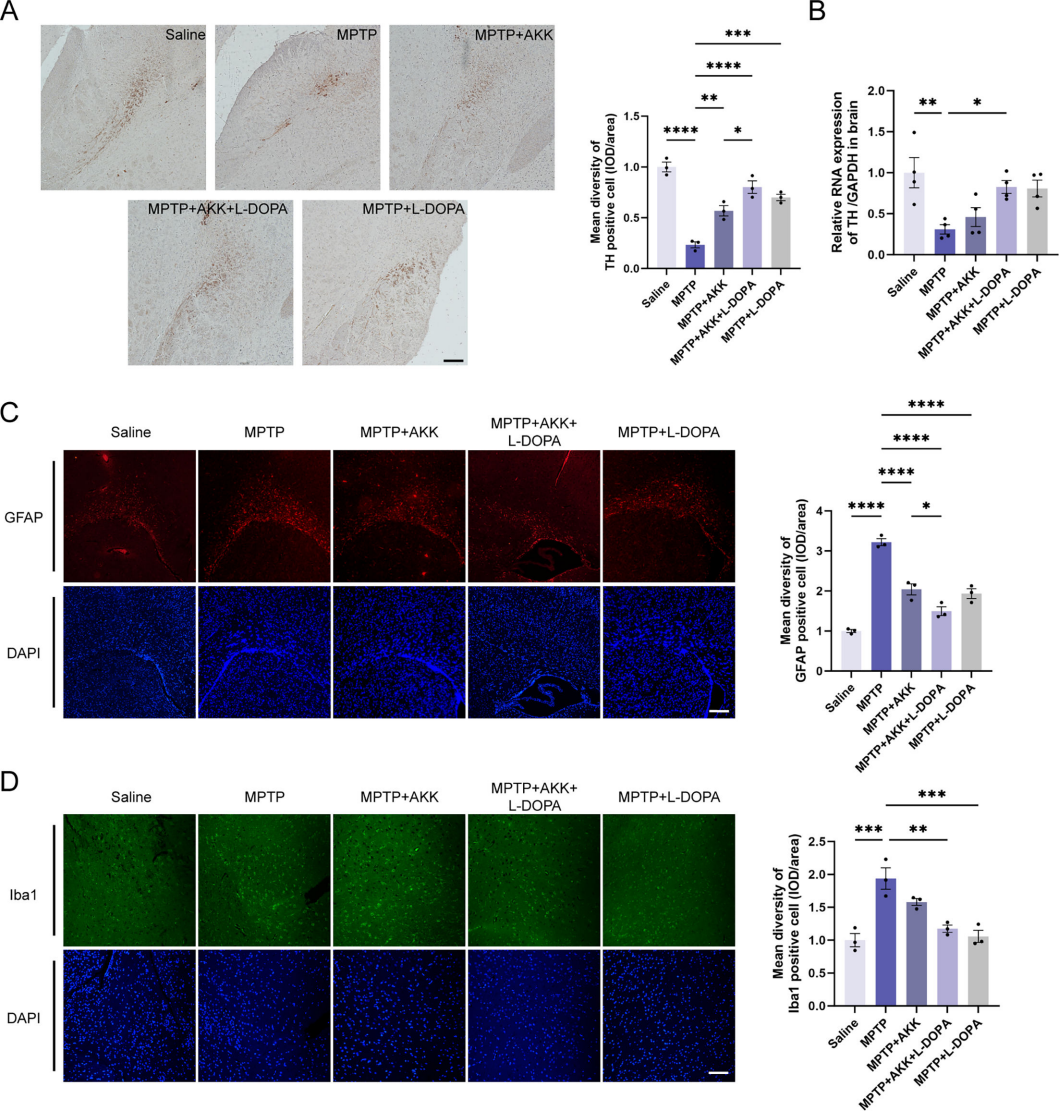
Investigation of the impact of hypoactive *Akkermansia muciniphila* on dopaminergic neurons and gliocytes in mice. (**A**) Immunohistochemical staining and quantification of tyrosine hydroxylase-positive cells in mice, along with the mean density of TH^+^ cells across different groups (*n* = 3). Scale bar = 100 gm. (**B**) Analysis of mRNA expression levels of TH. (**C**) Immunofluorescent staining and quantification of GFAP^+^ cells in mice, along with the mean density of GFAP^+^ cells across different groups (*n* = 3). Scale bar = 100 gm. (**D**) Immunofluorescent staining and quantification of Iba1^+^ cells in mice, along with the mean density of Iba1^+^ cells across different groups (*n* = 3). Scale bar = 100 gm. Data are presented as means ± SEM and were analyzed using one-way ANOVA followed by Tukey’s post hoc test. Statistical significance is indicated by *P < 0.05, **P < 0.005, ***P < 0.0005, and ****P < 0.0001. GFAP^+^, glial fibrillary acidic protein positive; Iba1^+^, ionized calcium-binding adapter molecule 1 positive.

The percentage of mice exhibiting positive tyrosine hydroxylase (TH) cell counts in the striatum of brain is depicted in Fig. 2A. Compared to the saline group, immunohistochemical analysis reveals that MPTP significantly decreases TH expression in the striatum of mice brain. This reduction is alleviated by treatment with hypoactive *Akkermansia muciniphila* and L-DOPA monotherapy, with the most pronounced effect observed when combined with L-DOPA. The mRNA and protein expression levels corroborate the immunohistochemical findings (Fig. 2B; Fig. S1); however, the therapeutic effect of hypoactive *Akkermansia muciniphila* appears limited, showing significant results only in conjunction with L-DOPA. Collectively, these findings suggest that hypoactive *Akkermansia muciniphila* mitigates MPTP-induced impairments in dopaminergic neurons in a pathological manner. This indicates that the synergistic interaction between hypoactive *Akkermansia muciniphila* and L-DOPA may represent a novel therapeutic strategy for the treatment of PD, potentially enhancing outcomes for patients suffering from dopaminergic neuronal degeneration.

### Hypoactive *Akkermansia muciniphila* reduces MPTP-induced glial reactivity and hardly provides neurotrophic and barrier-protected effect

**Fig 3:**
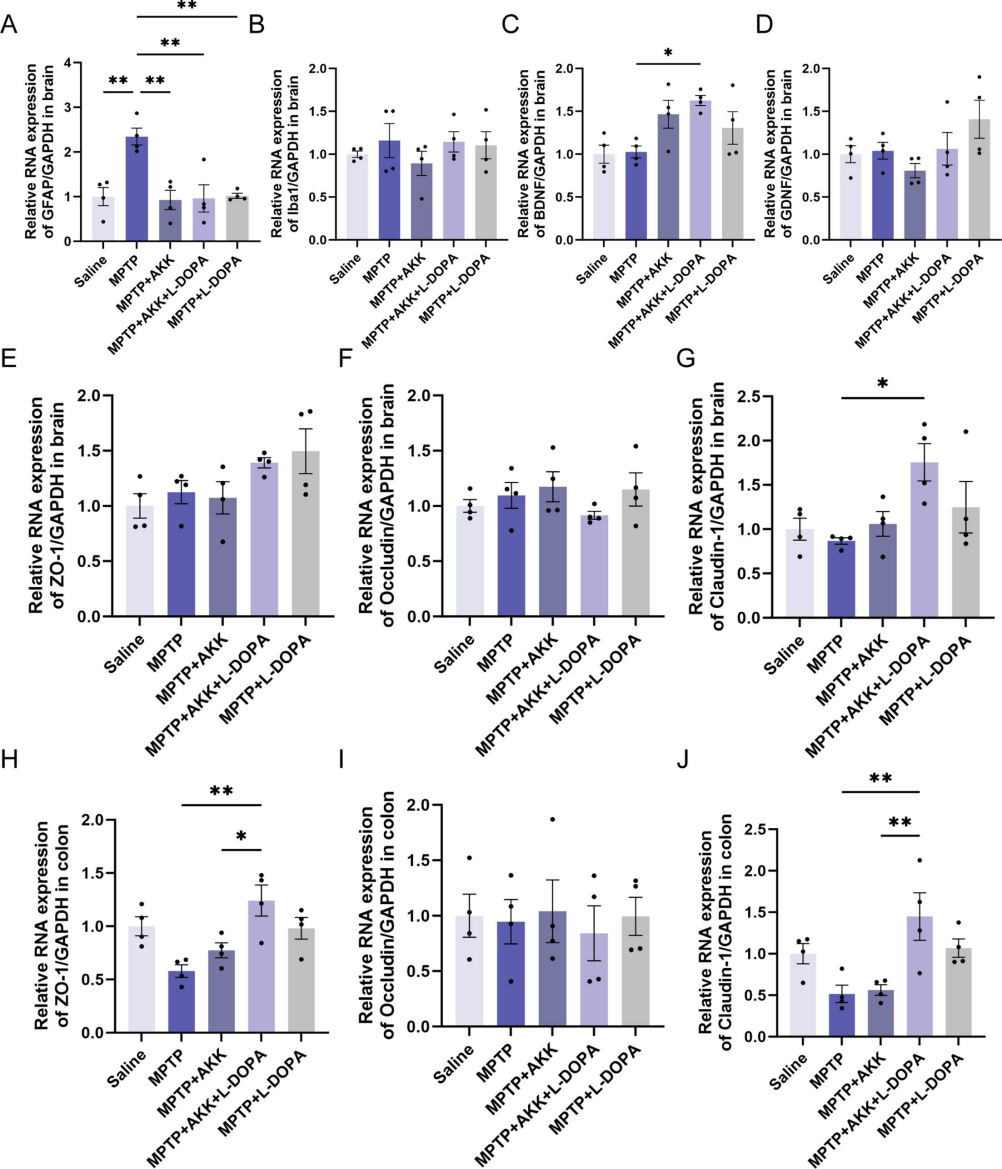
Investigation of the neuroprotective effects of hypoactive *Akkermansia muciniphila* and L-DOPA in the brain and colon. Panels **A** to **F** illustrate the mRNA expression levels of brain-derived neurotrophic factor (BDNF), Iba1, glial cell line-derived neurotrophic factor (GDNF), ZO-1, occludin, and claudin-1 in the brain (*n* = 4). Panels **G** to **J** depict the mRNA expression levels of ZO-1, occludin, and claudin-1 in colon (*n* = 4). Data are presented as means ± SEM and were analyzed using one-way ANOVA followed by Tukey’s post hoc test. Statistical significance is indicated by *P < 0.05 and **P < 0.005.

To investigate the neuroprotective effects of hypoactive *Akkermansia muciniphila*, we assessed whether its ingestion could modulate glial reactivity in a mouse model of PD induced by MPTP. Quantitative analysis of glial cell activation was performed by assessing the expression levels of two well-established neuroinflammatory markers, glial fibrillary acidic protein (GFAP) for astrocytes and ionized calcium-binding adapter molecule 1 (Iba1) for microglia, using RT-qPCR and immunofluorescence staining. GFAP levels were significantly elevated in the MPTP group compared to the saline group, whereas ingestion of hypoactive *Akkermansia muciniphila* effectively reduced GFAP levels. Notably, the combination therapy demonstrated significantly enhanced therapeutic efficacy compared to monotherapy approaches (Fig. 2C). Hypoactive *Akkermansia muciniphila* treatment also significantly downregulated GFAP mRNA expression, achieving comparable effects to L-DOPA monotherapy (Fig. 3A). Quantitative analysis also revealed significant upregulation of Iba1 expression following MPTP administration, indicating microglial activation. L-DOPA treatment significantly decreased Iba1 expression levels, whereas hypoactive *Akkermansia muciniphila* monotherapy failed to demonstrate significant microglial modulation (Fig. 2D). On the other hand, RT-qPCR analysis demonstrated comparable Iba1 mRNA expression among all treatment conditions, implying that microglial transcriptional regulation may involve other mechanisms (Fig. 3B).

We also examined the expression of brain-derived neurotrophic factor (BDNF) and glial cell line-derived neurotrophic factor (GDNF) in the brain. MPTP treatment did not significantly alter the mRNA levels of these neurotrophic factors in mice (Fig. 3C and D). Hypoactive *Akkermansia muciniphila* had minimal effects on BDNF levels, showing a significant increase only in conjunction with L-DOPA treatment (Fig. 3C). No significant differences in GDNF levels were observed among the experimental groups (Fig. 3D), in contrast to the expression observed for BDNF. This differential expression may be attributed to variations in the spatial distribution within the brain. Our modeling method only caused the initial symptoms of Parkinson’s disease but seemingly failed to cause damage to the blood-brain barrier (BBB). These findings suggest that hypoactive *Akkermansia muciniphila* may have limited neurotrophic effects. Furthermore, we investigated blood-brain and intestinal barrier function by examining the mRNA levels of three junction proteins: Zonula occludens-1 (ZO-1), claudin-1, and occludin. No significant differences were noted in the mRNA level of three junction proteins and ZO-1 protein level in the brain between the MPTP and saline groups (Fig. 3E through G; Fig. S2), indicating that MPTP treatment had not induced barrier damage in the brain in our experimental setup. The MPTP + AKK and MPTP + AKK + L-DOPA groups also showed no significant differences compared to the MPTP group (Fig. 3E and F), with the exception that claudin-1 levels were elevated in the hypoactive *Akkermansia muciniphila* combined with L-DOPA group (Fig. 3G), a similar result seen in the colon (Fig. 3J). Regarding the intestinal barrier, MPTP treatment decreased ZO-1 levels in the colon (Fig. 3H), and only the combinatorial therapy demonstrated a significant therapeutic effect. Few differences in occludin levels were observed between groups in both the brain and colon (Fig. 3F and I). In summary, whereas hypoactive *Akkermansia muciniphila* may not directly protect dopaminergic neurons through neurotrophic factors or barrier protection, it could potentially modulate the inflammatory response, thereby reducing glial reactivity and providing an alternative neuroprotective mechanism.

### Hypoactive *Akkermansia muciniphila* reduced MPTP-induced inflammation

**Fig 4:**
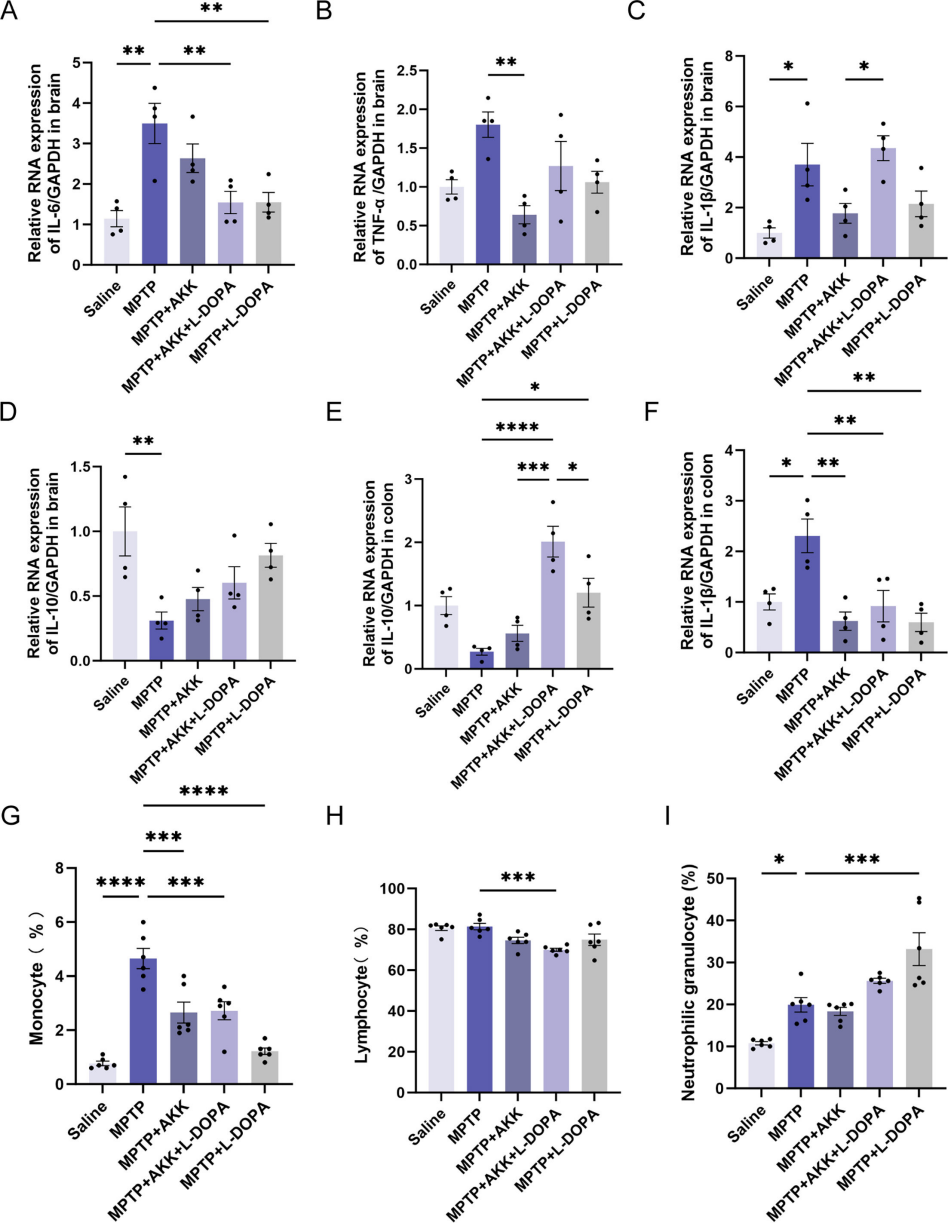
The effects of hypoactive *Akkermansia muciniphila* and L-DOPA on inflammation suppression. Panels **A** to **D** illustrate the mRNA expression levels of proinflammatory cytokines IL-6, TNF-a, IL-113, and the anti-inflammatory cytokine IL-10 in the brain (*n* = 4). Panels **E** and **F** present the mRNA expression levels of IL-113 and IL-10 in the colon (*n* = 4). Percentages of monocytes (**G**), lymphocytes (**H**), and neutrophilic granulocytes (**I**) are also depicted (*n* = 6). Data are presented as means ± SEM and were analyzed using one-way ANOVA followed by Tukey’s post hoc test. Statistical significance is indicated as follows: *P < 0.05, **P < 0.005, ***P < 0.0005, and ****P < 0.0001, in comparison to the MPTP group.

The levels of proinflammatory cytokines, including IL-6, TNF-α, and IL-1β, were measured in the brain (Fig. 4A through C). Results indicated that MPTP induction significantly increased the levels of TNF-α, IL-1β, and IL-6 in the brains of the MPTP group compared to the saline group. Ingestion of hypoactive *Akkermansia muciniphila* significantly reduced the expression level of IL-6 when combined with L-DOPA (Fig. 4A) and more effectively reduced the elevated TNF-α levels when combined with hypoactive *Akkermansia muciniphila* monotherapy (Fig. 4B). However, the IL-1β and TNF-α levels in the brains of the MPTP + AKK + L-DOPA group appeared to be elevated compared to the saline group (Fig. 4B and C). Furthermore, MPTP induction led to a significant decrease in the anti-inflammatory cytokine IL-10 in both the brain and colon, with hypoactive *Akkermansia muciniphila* having limited alleviative effects on these levels (Fig. 4D and E). MPTP toxicity also increased the IL-1β level in the colon, which was significantly reduced by hypoactive *Akkermansia muciniphila* (Fig. 4F). Blood routine tests revealed that MPTP induced increases in the percentages of neutrophilic granulocytes and monocytes, whereas hypoactive *Akkermansia muciniphila* influenced a reduction in monocyte (Fig. 4G) and eosinophilia percentages (Fig. S3A). The MPTP + AKK + L-DOPA group demonstrated a significant decrease in lymphocyte percentage concomitant with an elevated neutrophil granulocyte percentage (Fig. 4H and I). Meanwhile, we also observed that L-DOPA monotherapy significantly increased neutrophilic granulocyte percentage compared to MPTP groups (Fig. 4G). There was no significant difference among the remaining blood routine results of each group, and all these results were within the normal range (Fig. S3). Overall, hypoactive *Akkermansia muciniphila* may reduce MPTP-induced inflammation in the brain and colon by decreasing specific proinflammatory cytokines, though the effects appeared unstable when combined with L-DOPA. This suggests that whereas hypoactive *Akkermansia muciniphila* could serve as a potential therapeutic agent for mitigating MPTP-induced inflammation, further investigation is required to determine the efficacy and underlying mechanisms of its conjunction with L-DOPA. The findings highlight the complex interplay between microbiota and neuroinflammation, pointing to a novel therapeutic avenue that could potentially modulate the gut-brain axis.

### Hypoactive *Akkermansia muciniphila* and MPTP treatment effect on fecal microbiota composition

**Fig 5:**
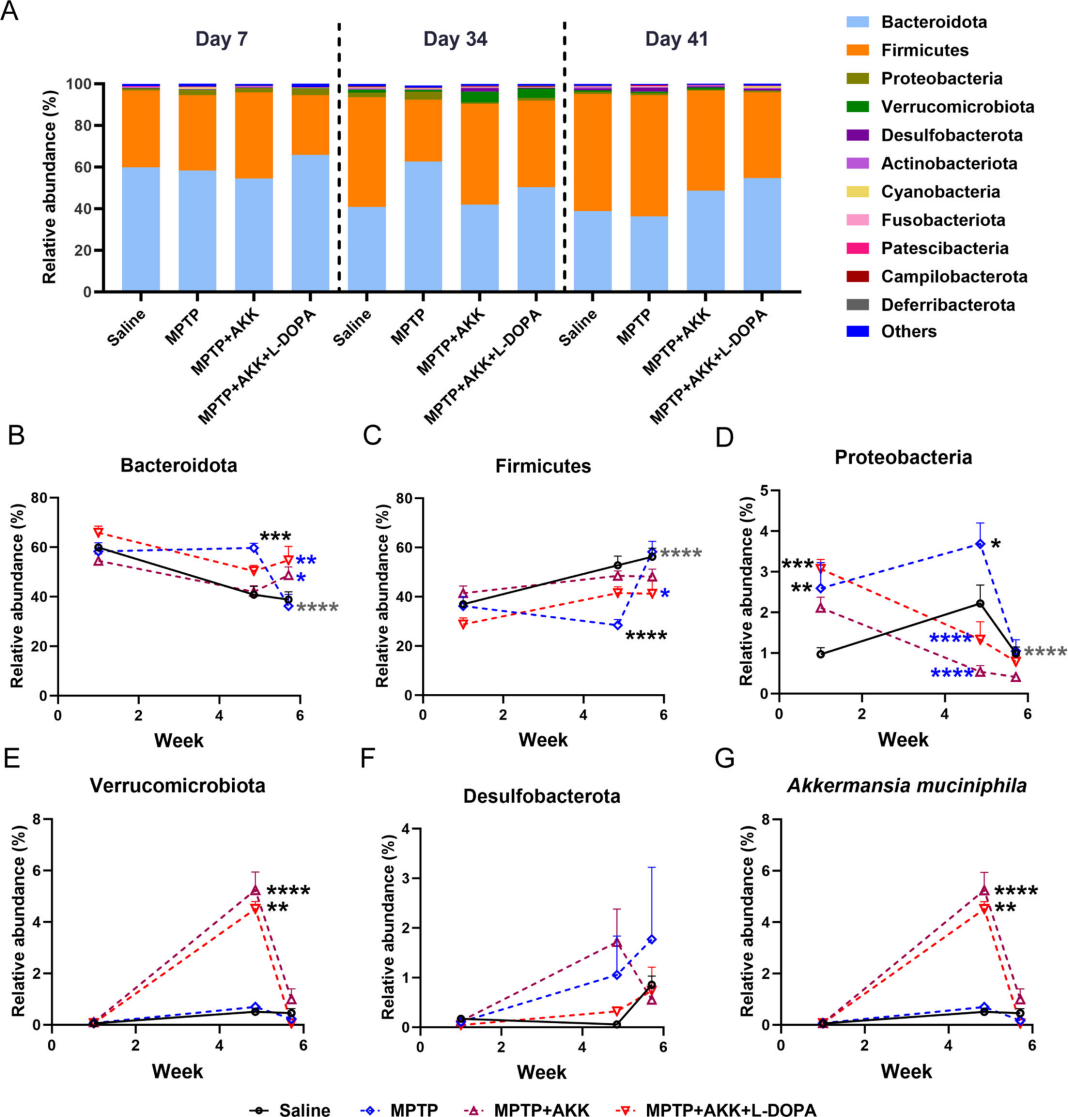
The fluctuations in the relative abundances of bacterial compositions in mouse fecal samples across various experimental time points. (**A**) A bar graph illustrates the relative compositions of the bacterial community at different time intervals (*n* = 6). The relative abundances of fecal (**B**) Bacteroidota, (**C**) Firmicutes, (**D**) Proteobacteria, (**E**) Verrucomicrobiota, (**F**) Desulfobacterota, and (**G**) *Akkermansia muciniphila* are presented for each group throughout the experimental period (*n* = 6). The data are presented as means ± SEM and were analyzed using two-way ANOVA followed by Tukey’s post hoc test and Dunn’s post hoc test. *P < 0.05, **P < 0.005, ***P < 0.0005 denote significant differences (*n* = 6). The blue/black asterisk indicates significant differences compared to the MPTP/saline group at each experimental time point; the gray asterisk indicates a significant difference within the same group between day 41 and day 34.

**Fig 6:**
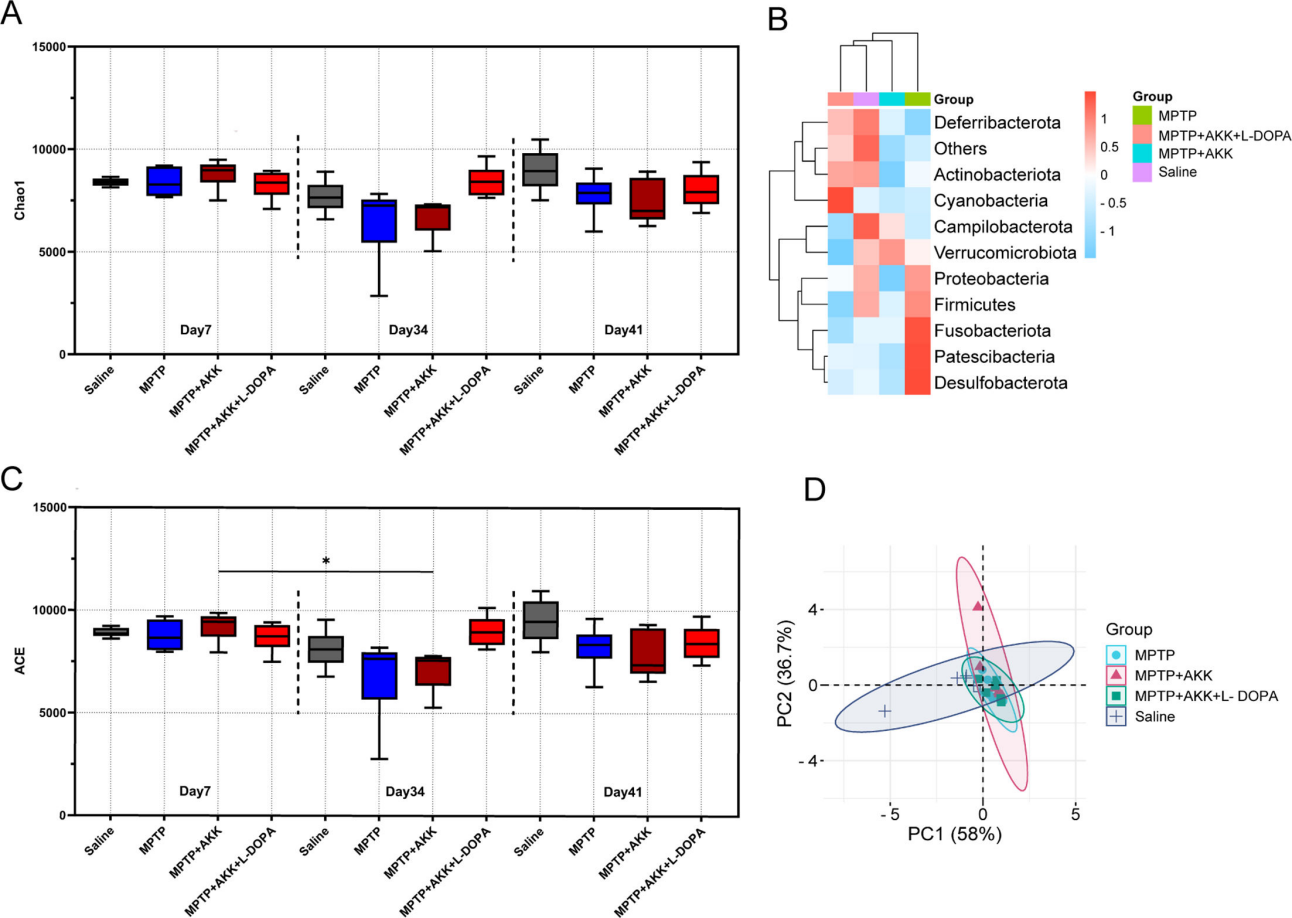
The administration of hypoactive *Akkermansia muciniphila* in conjunction with MPTP treatment significantly impacted the fecal microbiomes of mice. Variations in the diversity index among the groups were assessed using Chao1 (**A**) and ACE (**C**) estimator analyses (*n* = 6). The data are expressed as means ± SEM and were evaluated using the Kruskal-Wallis test followed by Dunn’s post hoc test. A *P*-value of *P < 0.05 denotes a statistically significant difference within the same group between day 41 and day 34. Panel **B** presents a heat map illustrating the relative abundance of bacterial phyla in mice on day 41 (*n* = 6). Panel **D** depicts the principal coordinate analysis of β-diversity, with axes labeled according to the contribution vectors (PCoA1 and PCoA2) of the principal components, and mouse samples are icon coded to distinguish between groups (*n* = 3).

We investigated the effects of experimental treatments on the mouse gut microbiota using 16S rRNA sequencing. Quantitative analysis confirmed a significant increase in *Akkermansia muciniphila* abundance following oral gavage administration. Baseline levels of *Akkermansia muciniphila* were detected in both saline group and MPTP group; these background levels did not significantly influence other experimental outcomes (Fig. 5G). At the phylum level, all experimental mice showed a significant increase in the relative abundance of Firmicutes and a corresponding decrease in Bacteroidetes on day 41 compared to day 7 (Fig. 5A through C). Feeding hypoactive *Akkermansia muciniphila* gradually resulted in a significant reduction in the population of Proteobacteria (*P* < 0.01) (Table S1) (Fig. 5D) and an increase in the population of Bacteroidetes in the MPTP + AKK and MPTP + AKK + L-DOPA groups after MPTP treatment (Fig. 5B). In contrast, the Bacteroidetes population significantly decreased (*P* < 0.0001) in the MPTP group (Fig. 5B). Additionally, when comparing day 41 to day 34, a significant increase in the Firmicutes/Bacteroidetes ratio was observed in the MPTP group (*P* < 0.01), whereas no significant changes were observed in other groups (Table S1). Both Verrucomicrobiota abundance and *Akkermansia muciniphila* levels significantly increased following oral gavage administration of hypoactive *Akkermansia muciniphila* and returned to baseline levels upon cessation of treatment (Fig. 5E and G; Fig. S4), confirming the controllability of hypoactive *Akkermansia muciniphila* supplementation. No significant differences in Desulfobacterota abundance were observed among the experimental groups, with all groups maintaining consistently low levels (Fig. 5F). The α-diversity analysis at the phylum level showed fluctuations in the MPTP + AKK groups from day 7 to day 34, as identified by the alternating conditional expectation (ACE) algorithm (Fig. 6A through C). The heatmap of phyla abundance indicated that the gut microbiota in the MPTP + AKK and MPTP + AKK + L-DOPA groups were more similar to the saline group than to the MPTP group (Fig. 6B). Furthermore, principal coordinate analysis (PCA) analysis revealed that the gut microbial community of the MPTP group was considerably different from that of the control group, whereas the microbial community of the MPTP + AKK and MPTP + AKK + L-DOPA groups was similar to that of the control group (Fig. 6D). Overall, the ingestion of *Akkermansia muciniphila* induced complex changes in gut microbiota composition, and its effects on PD mice may be driven by various microbial variations.

### Hypoactive *Akkermansia muciniphila* hardly affected the MPTP-induced SCFA changes

**Fig 7:**
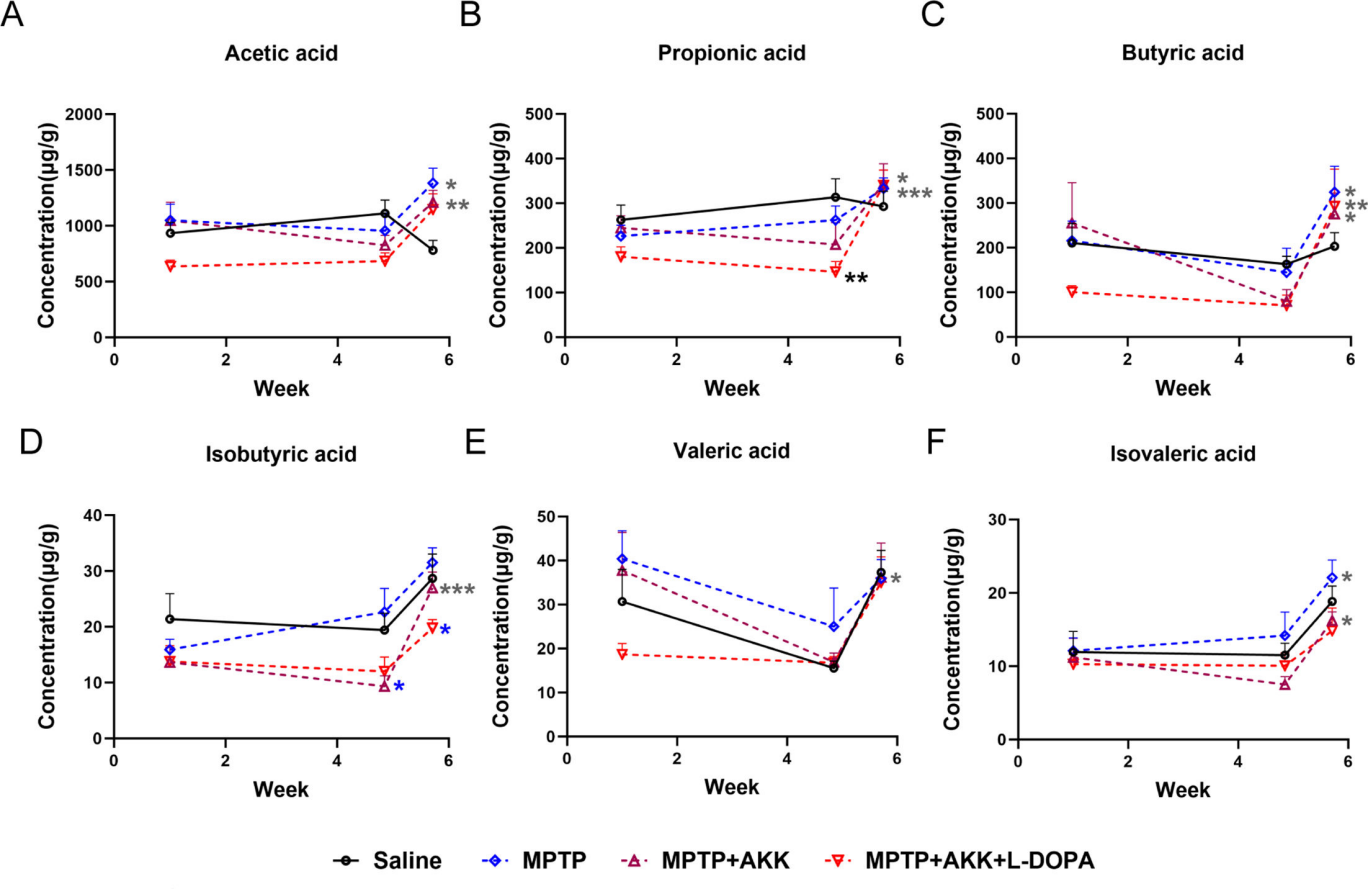
The fluctuations in fecal short-chain fatty acid concentrations across all mouse groups throughout the experimental period. The concentrations, measured in micrograms of SCFA per gram of feces (gg/g), included (**A**) acetic acid, (**B**) propionic acid, (**C**) butyric acid, (**D**) isobutyric acid, (**E**) valeric acid, and (**F**) isovaleric acid. Data are presented as means ± SEM and were analyzed using a two-way ANOVA followed by Tukey’s post hoc test. Statistical significance is indicated as follows: *P < 0.05, **P < 0.005, ***P < 0.0005 denote significant differences (*n* = 6); the blue/black asterisk indicates significant differences compared to the MPTP/saline group at each experimental time point; the gray asterisk indicates a significant difference within the same group between day 41 and day 34.

In the gut, SCFAs can be absorbed by intestinal epithelial cells and exert anti-inflammatory functions through various pathways. To test whether hypoactive *Akkermansia muciniphila* reduces MPTP-induced inflammation through SCFA pathway, we scrutinized the alterations in SCFA levels at each stage. Mouse feces from all experimental groups were collected on day 1 as the baseline, day 34 after the mice had received saline or hypoactive *Akkermansia muciniphila* (without MPTP treatment), and day 40 after MPTP or saline injection (Fig. 1A; Table S2). The acetic acid concentration in the MPTP group was significantly higher than in the saline group at day 40 (*P* < 0.05). Additionally, a notable increase in acetic acid concentration was observed in the MPTP + AKK group from day 34 to day 40 (*P* < 0.05) (Fig. 7A). An increase in the concentration of propionic acid (*P* < 0.01), butyric acid (*P* < 0.05), isobutyric acid (*P* < 0.001), and isovaleric acid (*P* < 0.05) was also observed in the MPTP + AKK group between day 34 and day 40 (Fig. 7B through E). Similar results were seen in the MPTP + AKK + L-DOPA group, where significant increases in propionic acid (*P* < 0.001) and butyric acid (*P* < 0.01) concentrations were found (Fig. 7B and C). No significant differences in isovaleric acid concentrations were observed among the experimental groups. However, all groups demonstrated a consistent increasing trend during the motor test period, potentially attributable to exercise-induced metabolic changes (Fig. 7F). The results indicated that the elevation of SCFAs across all groups was attributable to MPTP, rather than the influence of hypoactive *Akkermansia muciniphila*, suggesting that the hypoactive *Akkermansia muciniphila* does not mitigate inflammation via the SCFA pathway.

## DISCUSSION

In this study, we evaluated the adjunctive therapeutic potential of hypoactive *Akkermansia muciniphila* in alleviating the symptoms of PD in a mouse model induced by MPTP. Although hypoactive *Akkermansia muciniphila* showed minimal effects on motor deficits (Fig. 1) and the promotion of endogenous neuroprotective factors (Fig. 3) in the mouse brain, it did protect dopaminergic neurons (Fig. 2) and alleviate MPTP-induced neuroinflammation (Fig. 4). Additionally, the ingestion of hypoactive *Akkermansia muciniphila* influenced the concentrations of fecal short-chain fatty acids (Fig. 5) and modified gut microbiota composition, which may exert potential effects on PD therapy (Fig. 6).

MPTP administration is the most common method for creating a PD model without the need for sophisticated technology or specialized equipment. In our study, we utilized a well-established subacute dosing regimen developed by Tatton and Kish (35), which involves daily injections of 30 mg/kg MPTP for 5 days. This regimen is known to deplete striatal dopamine levels by approximately 40%–50% in mice, as documented in previous research. Although this approach effectively induces dopaminergic neuron degeneration, it has been noted in several studies that it often fails to elicit significant motor deficits (36, 37). Aside from some atypical symptoms such as piloerection immediately following injection, mice generally exhibit nearly normal behavior after MPTP administration. Our observations align with these findings: the majority of our mice displayed symptoms, including piloerection, arching of the back, tail erection, tremors, balance disorders, and salivation shortly after MPTP treatment. These effects persisted for approximately 20 minutes, after which the mice showed signs of fatigue and slowed respiration, with all symptoms resolving within 12 hours post-injection. Notably, weight loss was observed in most treatment groups, excluding the saline group, likely due to a decrease in food and water intake resulting from fatigue.

We conducted a second motor test 24 hours after the final MPTP injection but found no significant differences between the saline and MPTP groups. A comparative analysis of the pre- and post-MPTP treatment results revealed differences in the progression of mobility, as assessed by the PT and the NBT. Mice treated with MPTP exhibited reduced proficiency across multiple tests compared to untreated mice. The effects of hypoactive *Akkermansia muciniphila* and L-DOPA in this context were not noticeably pronounced and varied among individuals. The variability in improvement may be attributable to differences in the final concentrations of *Akkermansia muciniphila* and L-DOPA absorbed by the mice as well as their sensitivity to these substances. Incorporating a greater number of parallel samples could yield more robust results in this experiment, and conducting multiple tests would enable a more nuanced assessment of progression. Furthermore, refining the testing protocols—such as employing a modified rotarod test developed by Shiotsuki et al. (38)—could contribute to a more accurate evaluation of motor skill learning.

Cognitive dysfunction is one of the most prevalent non-motor symptoms in PD, impacting memory, attention, and executive as well as visual-spatial abilities (39). We evaluated the cognitive abilities of mice using the Y-maze test, which primarily assesses short-term memory and spatial cognition. The results revealed no significant differences between the saline- and MPTP-treated groups. We believe that the subacute dosing regimen is unlikely to produce cognitive impairment in mice due to the limited extent of impairment observed. Although the mechanisms underlying cognitive dysfunction in PD remain unclear, they are strongly associated with the disease’s progression over time (39, 40). Consequently, further studies are needed to elucidate the effects of hypoactive *Akkermansia muciniphila* on cognitive dysfunction in PD.

Research has demonstrated the involvement of inflammation in the onset and progression of PD, correlating with astrocytes, microglia, and endothelial cells (41). As the most abundant glial cell type in the brain, astrocytes play a crucial role in various CNS diseases. Typically, astrocytes are regarded as supportive cells involved in various functions, including participation in synaptic transmission (42) and managing the energy and redox requirements of neural activity (43). However, in response to trauma, infection, or neurodegenerative diseases, astrocytes can become reactive, leading to the upregulation of glial fibrillary acidic protein, a marker of reactive astrocytes (44). Reactive astrocytes exhibit protective effects following CNS insults by secreting neurotrophic factors, such as BDNF and GDNF (45). Conversely, certain types of reactive astrocytes have been found to exhibit neurotoxic properties (46), creating ambiguity about whether reactive astrocytes exert positive or negative effects in PD. Our results indicate astrocyte activation in MPTP-treated mice, which is inhibited by hypoactive *Akkermansia muciniphila*. We hypothesize that the inhibition of astrocyte activation may contribute to the therapeutic mechanisms of PD facilitated by hypoactive *Akkermansia muciniphila*.

Microglia, another type of glial cell, play a significant role in the pathophysiology of PD, particularly in relation to the cell-to-cell transfer of α-synuclein (47). Studies have indicated that the modulation of microglia in the substantia nigra is more closely associated with the degree of α-synuclein-induced pathology than to neuronal death (48). In our study, we did not observe any changes in the active microglia, marked by Iba1, induced by MPTP, nor did we detect any impact from hypoactive *Akkermansia muciniphila* on this process. We speculate that the lack of α-synuclein pathology generated by MPTP in our experiment may explain the absence of microglial activation. Additionally, other research has shown that MPTP fails to induce protein aggregation characteristic of Lewy bodies (49).

In terms of endothelial cells and the blood-brain barrier, patients with PD exhibit BBB leakage in the striatum (50), a condition also observed in MPTP-treated mice (51). Preventing damage to the BBB is essential for maintaining optimal nutrient supply and protecting the brain from harmful insults, representing a potential therapeutic strategy for PD. Our results demonstrated that hypoactive *Akkermansia muciniphila* did not enhance the expression of three barrier-related junction proteins: ZO-1, occludin, and claudin-1. Similar findings were reported for the intestinal barrier, suggesting that these junction proteins may not be the target of action for hypoactive *Akkermansia muciniphila*.

Inflammation contributes to the activation of glial cells, and barrier damage further exacerbates the inflammatory response, facilitating the diffusion of inflammatory factors and creating a vicious circle (41). High levels of inflammatory factors have been shown to lead to the degeneration of dopaminergic neurons by attracting peripheral inflammatory cells and increasing the release of pro-inflammatory cytokines and antibodies (52). In our experiments, we found that hypoactive *Akkermansia muciniphila* reduced MPTP-induced inflammation, potentially playing a crucial role in protecting dopaminergic neurons. The anti-inflammatory effects observed in the colon were consistent with findings from other studies involving live *Akkermansia muciniphila* (27, 53). The mechanism underlying the anti-inflammatory effect in the brain may be associated with the inhibition of astrocyte activation, as previously mentioned. Given the association between peripheral inflammatory cells and the degeneration of dopaminergic neurons, we conducted further blood analyses. Notably, the results showed an enrichment of classical monocytes in the peripheral blood of PD patients (54). Whether hypoactive *Akkermansia muciniphila* can reverse the dysregulation of monocytes warrants further investigation. Additionally, our study observed an abnormal elevation in the percentage of neutrophilic granulocytes when MPTP-induced mice were treated with a combination of hypoactive *Akkermansia muciniphila* and L-DOPA, which should be taken into consideration in future treatment research.

Gut microbiota composition may be influenced by various factors, including host age (55), diet (56), and disease (57). The microbial alterations observed in the gut differ among individuals with PD, but certain changes have been consistently confirmed. Research has identified several distinct taxa in the gut microbiota that differ significantly between individuals with PD and control subjects, particularly noting the prominence of Firmicutes and Bacteroidetes (58). Our results indicate that by day 41, compared to day 34, the abundance of Firmicutes and Bacteroidetes in the MPTP group exhibited significant fluctuations (Fig. 6A and B), leading to a marked increase in the Firmicutes/Bacteroidetes ratio (Table S1). This disruption in microbial balance may reflect the adverse effects induced by MPTP on gut microbiota. We also observed that treatment with both hypoactive *Akkermansia muciniphila* and MPTP resulted in the abundance of Firmicutes and Bacteroidetes being more closely aligned with that of the saline group. The stabilizing influence of hypoactive *Akkermansia muciniphila* on the gut microbiota was further validated through heatmap analysis. (Fig. 6B). Additionally, we observed a decrease in Proteobacteria in mice treated with hypoactive *Akkermansia muciniphila.* Proteobacteria have been found to be significantly more abundant in the mucosa of PD patients compared to controls (59); however, the impact of Proteobacteria depletion on PD progression or overall health remains unclear and requires further investigation. Although specific fluctuations in microbiota were noted following MPTP administration or treatment with hypoactive *Akkermansia muciniphila*, α-diversity analysis revealed that the diversity of gut microbiota remained relatively stable across different groups and time periods (Fig. 6A; Fig. S3). But a decrease in diversity was observed in the MPTP + AKK group by day 34 compared to day 1 when analyzed using the ACE algorithm, suggesting that hypoactive *Akkermansia muciniphila* retains its ability to influence gut microbiota diversity. Moreover, β-diversity analysis results further reveal the complex effects caused by the simultaneous presence of MPTP and hypoactive *Akkermansia muciniphila* (Fig. 6D). Overall, we discovered that both ingestion of hypoactive *Akkermansia muciniphila* and MPTP treatment induced fluctuations in gut microbiota. Further research is necessary to elucidate how these microbiota changes may produce either therapeutic or pathological effects.

SCFAs are crucial metabolites produced by gut bacteria, playing an essential role in maintaining intestinal homeostasis. In the gut, SCFAs can be absorbed by intestinal epithelial cells and exert anti-inflammatory functions through various pathways (60). Additionally, SCFAs influence the secretion of gut hormones by acting on enteroendocrine cells, signaling to the brain via hormonal transmission in systemic circulation or through vagal pathways (61). However, research on the direct effects of SCFAs on neurological diseases remains limited.

Our experiment showed no significant differences in SCFA concentrations among the groups during the same period. Nevertheless, we noted that hypoactive *Akkermansia muciniphila* is associated with more pronounced fluctuations in fecal SCFA levels, particularly when influenced by MPTP. Notably, the levels of propionic acid, butyric acid, isobutyric acid, and isovaleric acid increased due to the combined effects of hypoactive *Akkermansia muciniphila* and MPTP (Fig. 7B, C, D, and F). The results indicated that the elevation of SCFAs across all groups was attributable to MPTP, rather than the influence of hypoactive *Akkermansia muciniphila*, suggesting that the hypoactive *Akkermansia muciniphila* does not mitigate inflammation via the SCFA pathway.

### Conclusion

We have demonstrated that hypoactive *Akkermansia muciniphila* mitigates dopaminergic neuronal death, which correlates with a reduction in glial hyperactivation and neuroinflammation. Additionally, hypoactive *Akkermansia muciniphila* induced fluctuations in microbiota, potentially exerting complex effects on the progression of PD. Our study offers a novel strategy for utilizing hypoactive *Akkermansia muciniphila* either as a therapeutic agent or as an adjuvant therapy for PD.

## MATERIALS AND METHODS

### Animals and experimental groups

Seven-week-old male C57BL/6 mice were procured from Zhuhai Bestest Biotechnology Co., Ltd. (Zhuhai, China) and maintained under controlled environmental conditions at a temperature of 21°C–23°C, with a relative humidity of 50%–60% and a 12-hour light/dark cycle. Throughout the experimental period, the mice were provided with *ad libitum* access to food and water. The mice were randomly assigned to one of four groups (*n* = 10 mice per group): the saline group, the MPTP group, the MPTP + AKK group (treated with hypoactive *Akkermansia muciniphila*), and the MPTP + AKK + L-DOPA group (treated with L-DOPA plus benserazide). All experimental procedures involving animals were approved by the Laboratory Animal Welfare and Ethics Committee of Wuyi University, China (approval number: CN2023037).

The protocol for animal experimentation is illustrated in Fig. 1A. Mice were allowed a 1-week acclimatization period. From days 8 to 45, the MPTP + AKK and MPTP + AKK + L-DOPA groups received a daily oral gavage of *Akkermansia muciniphila* (10^8^ CFU/200 µL saline), while the saline and MPTP groups were administered saline in place of hypoactive *Akkermansia muciniphila*. During days 33–37, all groups except the saline group were administered MPTP daily (30 mg/kg intraperitoneal injection; Beyotime, Shanghai, China). Concurrently, the MPTP + AKK + L-DOPA group and MPTP + L-DOPA group received an additional oral gavage of L-DOPA (100 mg/kg; TargetMol) and benserazide (25 mg/kg; MedChemExpress). All mice were weighed every 3 days, and fecal samples were collected on days 7, 34, and 40. Furthermore, behavioral training was conducted on days 27–29, with testing occurring on days 30–34 and 41–45. All mice were sacrificed on day 46 for subsequent analysis.

### Hypoactive *Akkermansia muciniphila* preparation

*Akkermansia muciniphila* was procured from the Beijing Microbiological Culture Collection Center (Beijing, China). The freeze-dried strains were reactivated through anaerobic culturing in brain-heart infusion (BHI) broth (HuanKai Microbial, Guangdong, China) supplemented with 2 g/L mucin (Xushuo Biotechnology, Shanghai, China) at 37°C for 48 hours. Subsequently, the reactivated *Akkermansia muciniphila* was further cultured anaerobically in BHI broth at 37°C for an additional 48 hours, with subculturing occurring every 2 days. The bacterial cells, corresponding to the desired density, were harvested by centrifugation at 8,000 × *g* for 10 minutes, and the resulting bacterial pellets were stored at −80°C for up to 3 months. Prior to oral administration, the *Akkermansia muciniphila* pellets were resuspended in saline and thawed in a 37°C water bath.

### Behavioral tests for motor functions

Behavioral tests including pole test, narrow-beam test, and Y-maze. PT: Place a 50 cm vertical rod with a diameter of 1 cm and a rough surface into the cage. On each training day, the mice were first placed in the cage with a pole for environmental familiarization, then placed them on a pole 15 cm from the bottom of the cage three times, and then placed on poles 30 cm from the bottom of the cage and 50 cm from the top of the pole for training. On the day of the test, each mouse was placed on the top of the pole, and the time it took to descend to the bottom within 60 seconds was recorded. If the mouse fell or jumped off the pole within 60 seconds, the test was repeated. Each mouse was tested three times (1 minute interval between tests), and the average time was taken.

NBT: The beam apparatus comprises a plane measuring 50 cm in length and 0.8 cm in width, positioned at a height of 50 cm above the floor. A black box is located at one end of the beam, serving as the endpoint. During each training session, mice were initially placed in the black box for a 5-minute period of environmental acclimatization. Subsequently, they were positioned on the beam at a distance of 5 cm from the box and trained to traverse the beam to reach the box three times. The training progressed with beam distances of 15, 30, and 50 cm. On the testing day, each mouse was subjected to three trials on the 50 cm beam, with a 1-minute interval between trials. The time taken for each mouse to traverse the beam and enter the box was recorded, with a maximum allowable time of 60 seconds. If a mouse fell or returned to the starting point midway, the test was repeated. In cases where a mouse failed to reach the endpoint within 60 seconds or consistently fell off the beam, a time of 60 seconds was recorded. The average time across the three trials was calculated for each mouse.

Y-maze: The Y-maze utilized in this study has a single arm measuring 35 cm in length, 5 cm in width, and 20 cm in height. Prior to testing, each mouse is allowed a 30-minute acclimation period to familiarize itself with the environment. During the test, researchers remain present in the room with the lights on and maintain silence. The Y-maze is thoroughly cleaned and dried before each trial. The initial arm of the Y-maze is designated as arm B, while the other two arms are labeled A and C. Each mouse is placed in arm B, which is closest to the center of the Y-maze, and the timer is started. Mice are permitted to explore the Y-maze for a duration of 3 minutes. The starting position is recorded as B, and each time the mouse enters a new arm, its new position is documented. An entry is defined as the mouse having all four limbs within one of the arms. If a mouse remains in the same position for more than 60 seconds without exhibiting exploratory behavior, it is gently moved toward the center of the Y-maze, and the experiment continues. After testing each mouse, fecal matter is removed, and the maze is cleaned with alcohol. The next mouse is introduced into the maze only after it is completely dry. Spontaneous alternation and exploratory behavior are quantified, with exploratory behavior calculated as the total number of entries within the 3-minute period.

### Sample collection and tissue preparation

Fecal samples were collected in individual sterile tubes and stored at −80°C until further analysis. Following the euthanasia of the mice, brain, colon, and blood samples were obtained. Blood samples were collected into tubes containing EDTA anticoagulant and kept on ice. The brains and colons were rapidly dissected, rinsed with saline, and immediately stored at −80°C for subsequent quantitative real-time PCR and western blot analysis. Protein concentrations in the mouse tissues were quantified using the Bradford assay (Beyotime, Shanghai, China). For immunohistochemical staining, whole brains were extracted and immersed in a 4% paraformaldehyde solution.

### Blood analysis

In accordance with the operator’s manual for the Auto Hematology Analyzer (Mindray Animal Medical Technology, Shenzhen, China), blood analysis was conducted utilizing whole blood samples. For the analysis, which was specifically calibrated for murine subjects, a volume of 15 µL of blood was required per sample.

### Quantitative real-time polymerase chain reaction

In accordance with the established protocol utilizing total RNA extraction reagents (Solarbio, Beijing, China), RNA was isolated from both brain and colon tissues. The isolated RNA was subsequently reverse transcribed into complementary DNA (cDNA) using the First-Strand cDNA Synthesis Kit (Codonx, Beijing, China). Quantitative PCR was performed employing the 2xSYBR Green qPCR Master Mix (Selleck, USA) on a BioRad-CFX96 system (Bio-Rad, California, USA). The cycle threshold (Ct) values were determined and normalized using the 2^−ΔCt^ method relative to GAPDH, to assess the relative gene expression levels. All primers were listed in Table S3.

### Western blot

For the western blot analysis, equivalent quantities of protein were loaded and separated on 15% SDS-PAGE gels, followed by transfer to PVDF membranes (Roche) via electrophoresis. The membranes were subsequently blocked for 1 hour using a blocking buffer composed of 5% skim milk in Tris-buffered saline with 0.1% Tween-20 (TBST). The membranes were then sectioned according to the size of the target proteins. The sections containing the target protein were incubated with the anti-TH antibody (1:1,000; Cell Signaling, Danvers, MA, USA) or β-actin antibody (1:5,000; GeneTex, USA). Following two washes with TBST, the membranes were incubated with a secondary antibody (goat anti-mouse IgG, 1:2,000; Zenbio, USA). Image analysis was conducted using ImageJ software. Protein expression levels were quantified as fold changes relative to the saline group, normalized against β-actin.

### Immunohistochemistry

Brain tissue was treated according to processed following standard paraffin section immunohistochemistry experimental procedures. Sections were blocked by 5% protocols. The sections were blocked with 5% bovine serum albumin (Beyotime, Shanghai, China) and incubated with a mouse anti-TH-tyrosine hydroxylase antibody (1:400, Cell Signaling, Danvers, MA, USA). Afterward, the sections were incubated with a secondary antibody (goat anti-mouse IgG, 1:200, Zenbio) incubation. 3,3-Diaminobenzidine (Solarbio, Beijing, China) staining was then used to visualize substantia nigra pars compacta TH-immunoreactive neurons, next with re-staining of the nuclei, and then followed by nuclear counterstaining. The sections were then dehydrated and sealed. We used a microscope (Nikon, Japan) to picture the stained slides. Microscopic imaging was performed on the stained slides, and then ImageJ was utilized for subsequent analysis.

### Fecal SCFA analysis

Fecal samples were homogenized in a 20% phosphoric acid buffer containing 4-methyl valeric acid as a surrogate standard, followed by vortexing for 2 minutes and centrifugation at 14,000 × *g* for 20 minutes. The resulting supernatant was collected into sample vials for subsequent gas chromatography-mass spectrometry (GC-MS) analysis. The GC-MS system utilized was an Agilent 7890B gas chromatograph coupled with an Agilent 5977B chemical ionization/electron ionization mass selective detector (Agilent Technologies, Santa Clara, CA, USA), an Agilent 7683 automatic liquid sampler, and a fused-silica capillary column with a free fatty acid phase, measuring 30 m × 250 μm × 0.25 µm (DB-FFAP, J&W Scientific, Agilent Technologies). A 1 µL injection was carried out in splitless mode at 250°C. The initial column temperature was set at 90°C, which was increased to 160°C at a rate of 10°C per minute, followed by a further increase to 240°C at a rate of 40°C per minute, and maintained for 5 minutes. Helium served as the carrier gas at a flow rate of 1.0 mL/min with a split ratio of 10:1. The ion source, quadrupole, and interface temperatures were maintained at 230°C, 150°C, and 250°C, respectively. The detector operated in SCAN/SIM mode, targeting the detection of acetic, propionic, isobutyric, butyric, isovaleric, and valeric acids.

### Gut microbial and bioinformatics analysis

The methodology for sequencing fecal 16S rRNA adhered to established protocols as outlined in reference (35). Operational Taxonomic Unit (OTU) clustering was conducted on unique sequences, defined as those with more than one occurrence, based on a 97% similarity threshold. Sequences exhibiting greater than 97% similarity to the representative OTU sequences were classified as the same OTU. The R programming language was employed to generate a clustering heat map, illustrating the distribution of the top 10 species at the phylum level within each group. This visualization effectively highlights the similarities and differences among species through color coding. The estimation of OTU numbers within the samples was carried out using the Chao1, ACE, Shannon, and Simpson indices, which served to reflect the α-diversity index and provide an assessment of microbial diversity within a sample. β-Diversity was calculated utilizing Bray-Curtis distances and was visualized through PCA.

### PCR and agarose gel electrophoresis

Extract genomic DNA from feces using TIANamp Stool DNA Kit (TIANGEN BIOTECH, Beijing) and take 1 µL of the product for PCR. The primer sequences are shown in Table S3. Take the bacterial culture containing 10^8^ CFU *Akkermansia muciniphila*, extract the bacterial genome using the TIANamp Bacteria DNA Kit (TIANGEN BIOTECH, Beijing), and the resulting product serves as the positive control. PCR was performed employing Ex TaqTM Version 2.0 (Takara, Japan) on the C1000 Touch PCR thermal cycler (Bio-Rad, California, USA). Furthermore, 1% agarose gel was prepared using agarose and TAE buffer. The PCR product (1 µL) was mixed with loading buffer and loaded into the sample wells. Electrophoresis was conducted at a constant voltage of 120 V for 25 minutes. Finally, the gel was visualized using a gel imaging system.

### Statistical analysis

Statistical analyses were performed utilizing GraphPad Prism version 10 software. Data are expressed as mean ± SEM. Intergroup differences were assessed through one-way ANOVA with Tukey’s post hoc test or Kruskal-Wallis test followed by Dunn’s post hoc test. Differences among groups within the same period and across different periods within the same group were assessed through two-way ANOVA. The *P*-value of less than 0.05 was deemed to indicate statistical significance.

## Data Availability

The original contributions presented in the study are publicly available. The data can be found under PRJNA1242010.

## SUPPLEMENTAL MATERIAL

Fig. S1 spectrum.03379-24-s0001.tif Western blot analysis and quantification of TH expression levels in mice.

Fig. S2 spectrum.03379-24-s0002.tif The effects of hypoactive *Akkermansia muciniphila* and MPTP on blood routine of mice.

Fig. S3 spectrum.03379-24-s0003.tif Immunohistochemical staining and quantification of ZO-1 tight junction protein in brain, along with the mean density across different groups (expressed as fold changes compared with the Saline group, n = 3).

Fig. S4 spectrum.03379-24-s0004.png PCR showed the content of *Akkermansia muciniphila* in feces at different time points.

Table S1 spectrum.03379-24-s0005.docx Phyla with relative abundances greater than 0.5% of the total population are presented.

Table S2 spectrum.03379-24-s0006.docx Short-chain fatty acid (SCFA) concentrations exceeding 10 μg/g in mouse feces are reported.

Table S3 spectrum.03379-24-s0007.docx Primers information used for qRT-PCR.

ASM does not own the copyrights to Supplemental Material that may be linked to, or accessed through, an article. The authors have granted ASM a non-exclusive, world-wide license to publish the Supplemental Material files. Please contact the corresponding author directly for reuse.
