# Supplementary figures and images for: Neuroprotective effects of hypoactive Akkermansia muciniphila in MPTP-induced mouse models of Parkinson’s disease
Source: Microbiol Spectr. 2025 Nov 12;13(12):e03379-24. doi: 10.1128/spectrum.03379-24 (PMC12671141; doi:10.1128/spectrum.03379-24)

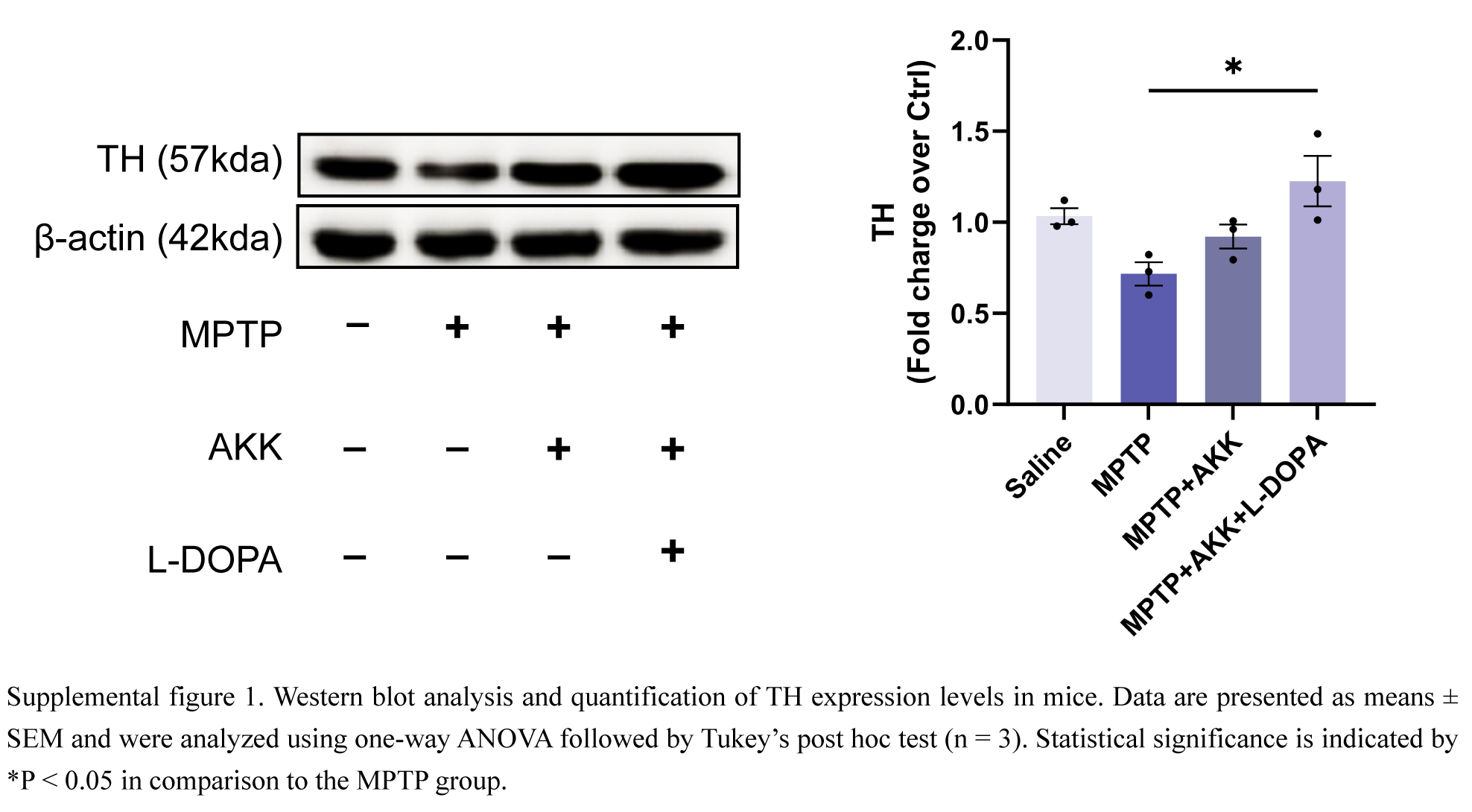

Supplement: Fig. S1 — Western blot analysis and quantification of TH expression levels in mice. [file spectrum.03379-24-s0001.tif]

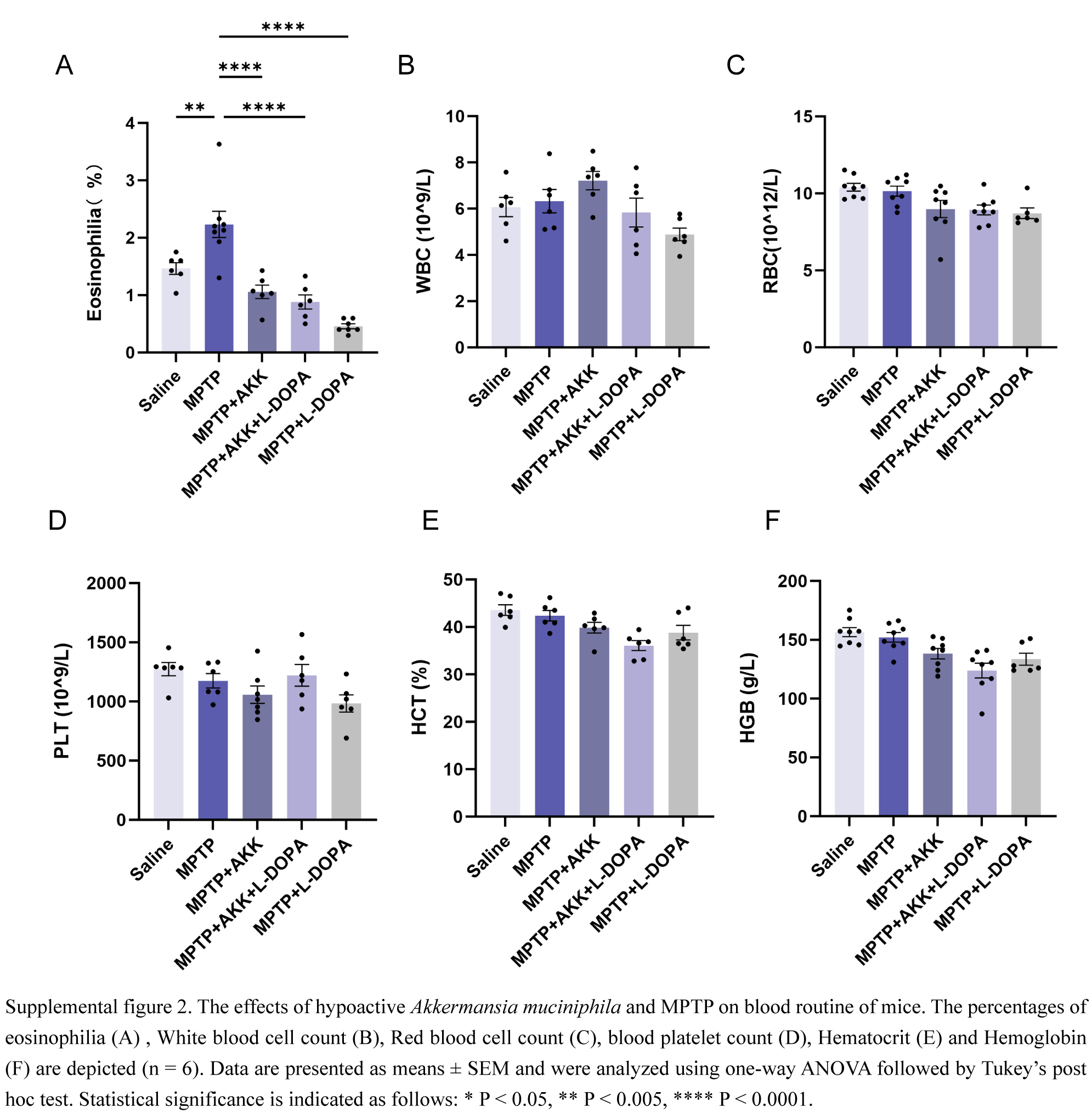

Supplement: Fig. S2 — The effects of hypoactive Akkermansia muciniphila and MPTP on blood routine of mice. [file spectrum.03379-24-s0002.tif]

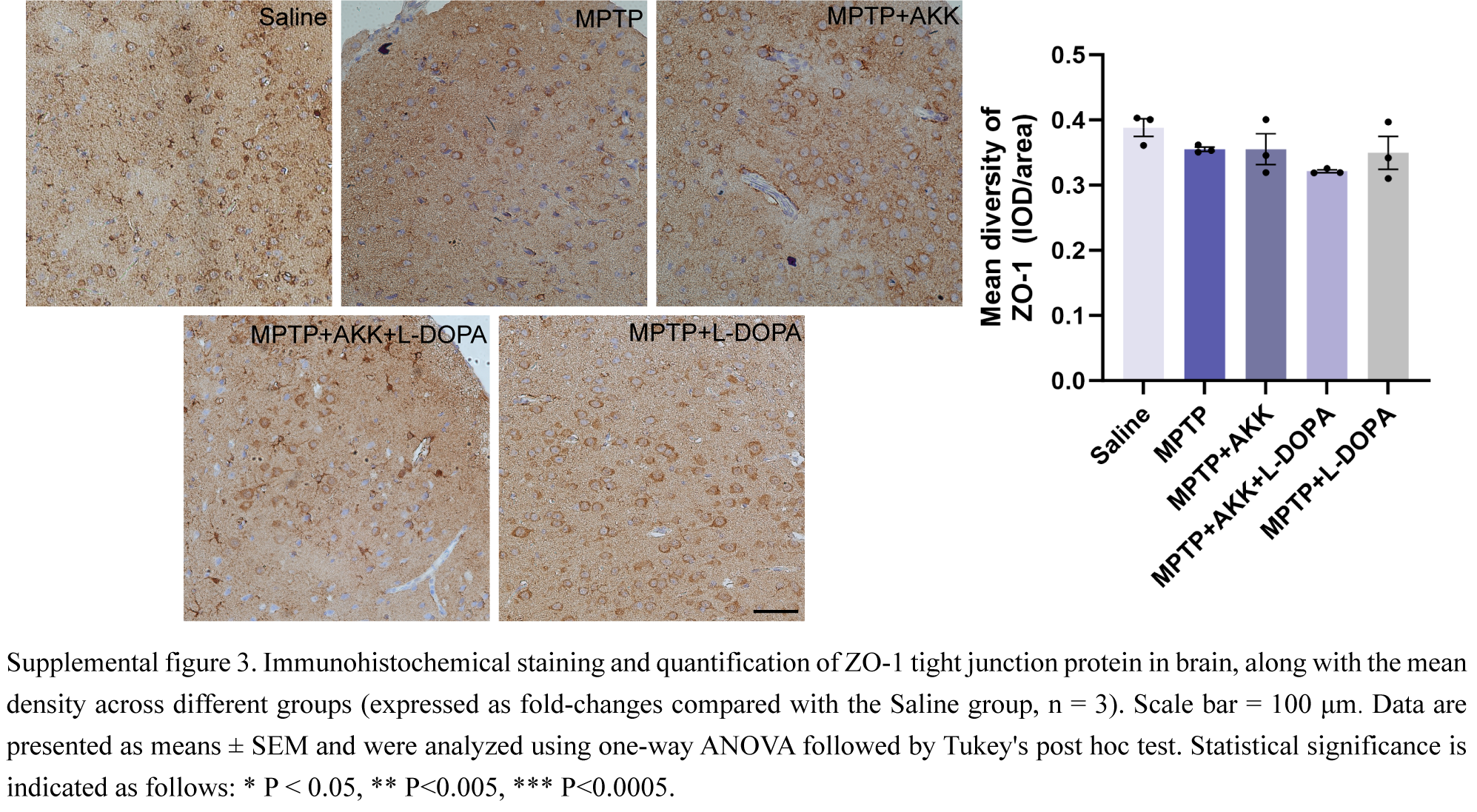

Supplement: Fig. S3 — Immunohistochemical staining and quantification of ZO-1 tight junction protein in brain, along with the mean density across different groups (expressed as fold changes compared with the Saline group, n = 3). [file spectrum.03379-24-s0003.tif]

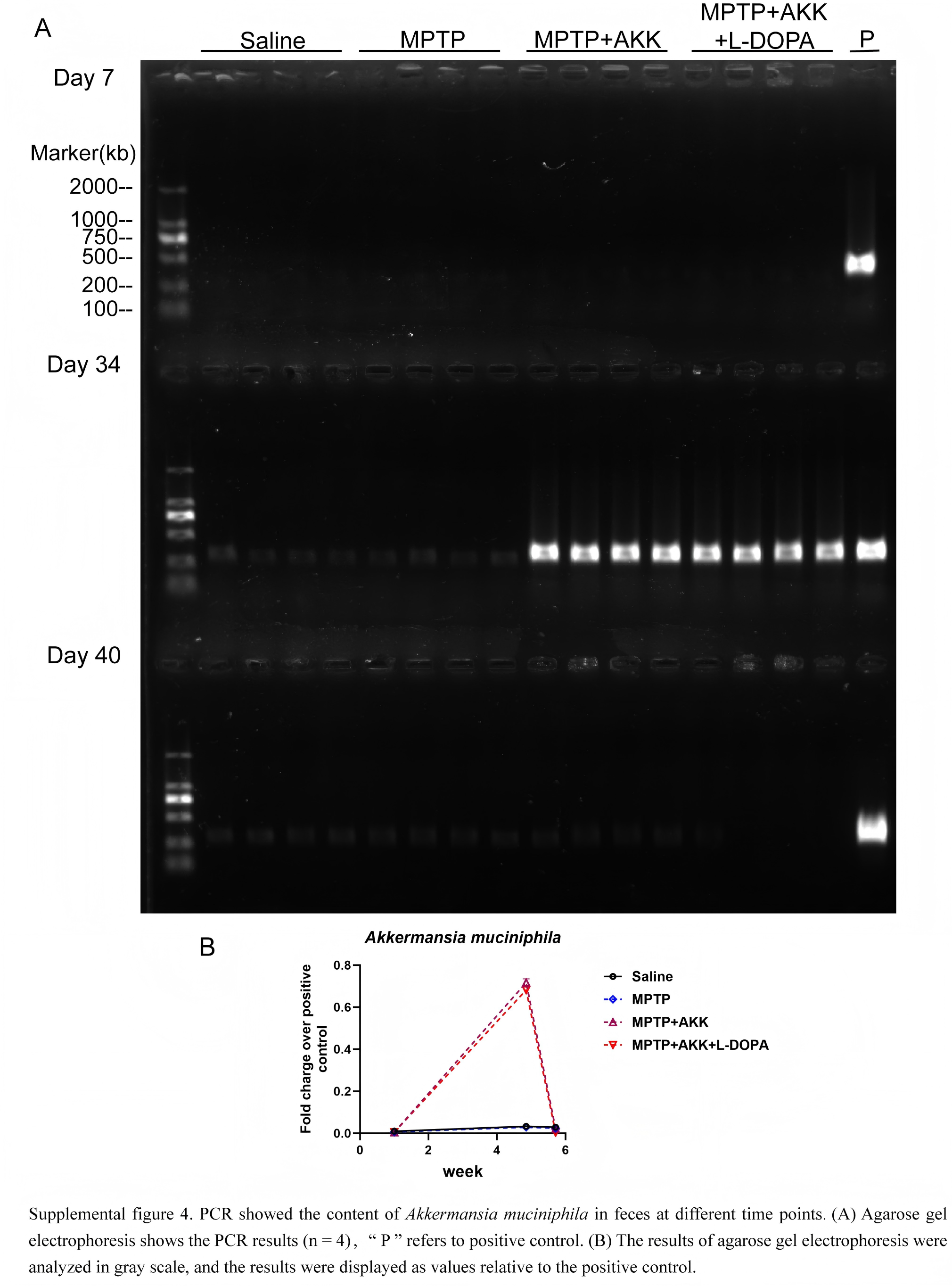

Supplement: Fig. S4 — PCR showed the content of Akkermansia muciniphila in feces at different time points. [file spectrum.03379-24-s0004.png]
