# Supplementary material for: Neuroprotective effects of hypoactive Akkermansia muciniphila in MPTP-induced mouse models of Parkinson’s disease
Source: Microbiol Spectr. 2025 Nov 12;13(12):e03379-24. doi: 10.1128/spectrum.03379-24 (PMC12671141; doi:10.1128/spectrum.03379-24)
Supplement: Table S1 — Phyla with relative abundances greater than 0.5% of the total population are presented. [file spectrum.03379-24-s0005.docx]

| **Supplemental Table 1. Relative abundances of phylum-level bacterial compositions in mouse fecal samples and ratios of bacterial communities at different experimental times.** | | | | | | | | | | | | |
| --- | --- | --- | --- | --- | --- | --- | --- | --- | --- | --- | --- | --- |
| Day | Day 7 | | | | Day 34 | | | | Day 41 | | | |
| Group | Saline | MPTP | MPTP+AKK | MPTP+  AKK+L-DOPA | Saline | MPTP | MPTP+AKK | MPTP+  AKK+L-DOPA | Saline | MPTP | MPTP+AKK | MPTP+  AKK+L-DOPA |
| Phylum level (relative abundance (%)) | | | | | | | | | | | | |
| Bacteroidota | 59.9±2.0% | 58.3±7.8% | 54.5±6.2% | 65.8±5.9% | 40.8±7.5% | 59.7±4.1%^***^ | 42.0±5.5%^$$^ | 50.3±5.1% | 38.9±7.0% | 36.3±10.4%^####^ | 48.7±7.4%^$^ | 54.7±12.6%^**$$^ |
| Firmicutes | 36.9±1.4% | 36.2±8.7% | 41.4±6.5% | 28.7±5.9%^$^ | 52.8±8.3% | 28.4±5.2%^****^ | 48.5±4.5%^$$$^ | 41.5±5.7%^$^ | 56.3±7.6% | 58.3±9.5%^####^ | 48.1±7.0% | 41.3±12.5%^$$^ |
| Proteobacteria | 1.0±0.4% | 2.6±1.4%^**^ | 2.1±0.6% | 3.1±0.5%^***^ | 2.2±1.0% | 3.7±1.1%^*^ | 0.5±0.3%^**$$$$^ | 1.3±1.0%^$$$$^ | 1.0±0.4%^#^ | 1.0±0.6%^####^ | 0.4±0.1% | 0.8±0.3% |
| Verrucomicrobiota | 0.1±0.02% | 0.1±0.02% | 0.1±0.02% | 0.1±0.04% | 1.4±1.1% | 5.4±3.7%^****^ | 5.2±1.6%^****^ | 4.5±0.6%^**^ | 0.8±0.8% | 0.7±1.0%^####^ | 1.0±0.9%^####^ | 0.1±0.03%^####^ |
| Desulfobacterota | 0.2±0.1% | 0.1±0.1% | 0.1±0.1% | 0.0±0.02% | 0.1±0.01% | 1.1±1.7% | 1.7±1.5% | 0.3±0.2% | 0.9±0.4% | 1.8±3.2% | 0.6±0.5% | 0.7±1.1% |
| Others | 1.0±0.2% | 1.2±0.4% | 1.0±0.2% | 1.6±0.5%^**$^ | 1.0±0.2% | 0.7±0.3% | 0.8±0.05% | 0.9±0.1% | 0.9±0.1% | 0.6±0.3% | 0.5±0.1%^*^ | 0.8±0.1% |
| Bacterial communities ratios | | | | | | | | | | | | |
| F/B ratio | 0.6±0.04 | 0.5±0.1 | 0.8±0.2 | 0.4±0.1 | 1.4±0.5 | 0.7±0.2 | 1.2±0.2 | 0.8±0.2 | 1.5±0.5 | 1.8±0.8^###^ | 1.0±0.3 | 0.9±0.5^$$^ |

Phyla with relative abundances greater than 0.5% of the total population are presented. The *Firmicutes/Bacteroidetes* (F/B) ratio is also provided. Data are expressed as means ± standard error of the mean (S.E.M.) and were analyzed using a two-way analysis of variance (ANOVA) followed by Tukey’s post hoc test, and subsequently by Dunn’s post hoc test. Significance levels are indicated as follows: ####P < 0.0001 for comparisons within the same group between day 41 and day 34; *P < 0.05, **P < 0.005, ****P < 0.0001 for comparisons with the Saline group at each experimental time point (n = 6); %%%%P < 0.0001 for comparisons within the same group between day 34 and day 1; $P < 0.05, $$P < 0.005, $$$$P < 0.0001 for comparisons with the MPTP group at each experimental time point (n = 6).
