# Supplementary material for: Neuroprotective effects of hypoactive Akkermansia muciniphila in MPTP-induced mouse models of Parkinson’s disease
Source: Microbiol Spectr. 2025 Nov 12;13(12):e03379-24. doi: 10.1128/spectrum.03379-24 (PMC12671141; doi:10.1128/spectrum.03379-24)
Supplement: Table S2 — Short-chain fatty acid (SCFA) concentrations exceeding 10 μg/g in mouse feces are reported. [file spectrum.03379-24-s0006.docx]

| **Supplemental Table 2. Different SCFAs concentration in mouse fecal samples.** | | | | | | | | | | | | | | |
| --- | --- | --- | --- | --- | --- | --- | --- | --- | --- | --- | --- | --- | --- | --- |
| Day | Day 7 | | | | Day 34 | | | | Day 41 | | | | | |
| Group | Saline | MPTP | MPTP+AKK | MPTP+  AKK+L-DOPA | Saline | MPTP | MPTP+AKK | MPTP+  AKK+L-DOPA | Saline | MPTP | MPTP+AKK | MPTP+  AKK+L-DOPA |  |  |
| SCFA concentration (μg/μl) | | | | | | | | | | | | | | |
| Acetic acid | 933.5±219.3 | 1049.6±322.6 | 1048.1±363.1 | 635.1±127.3 | 1110.2±270.3 | 956.6±353.0 | 825.2±199.5 | 684.6±164.0 | 780.0±198.4 | 1715.8±638.2^*#^ | 1210.7±244.2^##^ | 1147.6±314.0 |  |  |
| Propionic acid | 262.6±74.7 | 226.7±54.0 | 244.7±60.8 | 180.2±49.0 | 313.6±92.2 | 262.5±70.1 | 207.8±108.3 | 146.3±51.8^**^ | 292.8±75.9 | 333.1±53.4 | 343.6±100.0^#^ | 340.2±76.6^###^ |  |  |
| Butyric acid | 210.6±97.8 | 215.5±97.8 | 255.1±202.5 | 100.8±31.1 | 163.2±39.2 | 144.8±119. 8^#^ | 79.9±58.9^#%^ | 70.8±51.8^##^ | 203.3±67.5 | 324.4±129.7^#^ | 275.5±105.9^#^ | 292.6±3.4^##^ |  |  |
| Isobutyric acid | 21.4±10.2 | 15.9±4.1 | 13.7±5.9 | 13.8±6.3 | 19.4±6.7 | 22.7±9.4 | 9.3±4.2^$^ | 12.0±5.7 | 28.7±9.7 | 31.5±5.9 | 27.0±6.3^###^ | 19.8±3.4^$^ |  |  |
| Valeric acid | 30.7±16.3 | 40.4±14.2 | 37.8±19.3 | 18.7±5.5^$^ | 15.5±3.6 | 25.0±19.6 | 16.9±4.6^%^ | 16.8±3.2 | 37.3±11.2^#^ | 35.9±9.6 | 36.2±17.4 | 35.0±13.0 |  |  |
| Isovaleric acid | 11.9±6.3 | 12.1±3.9 | 11.2±5.8 | 10.3±3.0 | 11.5±3.6 | 14.2±7.1 | 7.5±2.4 | 10.1±4.0 | 18.8±4.7 | 22.1±5.4 | 16.2±2.6^#^ | 14.8±6.9 |  |  |

Short-chain fatty acid (SCFA) concentrations exceeding 10 μg/g in mouse feces are reported. The data are presented as means ± standard error of the mean (SEM) and were analyzed using a two-way analysis of variance (ANOVA) followed by Tukey's post hoc test. Statistical significance is indicated as follows: #P < 0.05, ##P < 0.01, ###P < 0.001, for comparisons within the same group between day 41 and day 34; *P < 0.05, **P < 0.01, for comparisons with the Saline group at each experimental time point (n = 6); %P < 0.05, for comparisons within the same group between day 34 and day 1; and $P < 0.05, for comparisons with the MPTP group at each experimental time point (n = 6).
