# Supplementary material for: Neuroprotective effects of hypoactive Akkermansia muciniphila in MPTP-induced mouse models of Parkinson’s disease
Source: Microbiol Spectr. 2025 Nov 12;13(12):e03379-24. doi: 10.1128/spectrum.03379-24 (PMC12671141; doi:10.1128/spectrum.03379-24)
Supplement: Table S3 — Primers information used for qRT-PCR. [file spectrum.03379-24-s0007.docx]

| **Supplemental Table 3. Primers information used for qRT-PCR** | | | | |
| --- | --- | --- | --- | --- |
| Primer | GenBank Accession | Sequence (5'-3') | Location | Amplicon Size (bp) |
| TH | NM_009377 | CCCAAGGGCTTCAGAAGAG  GGGCATCCTCGATGAGACT | 65-83  170-152 | 106 |
| GFAP | NM_001131020 | TCTGCCGTCCAAACTTGAAGCC  CTCTTCAGCTCTAGGTGGGTCT | 443-464  580-559 | 138 |
| Iba1 | NM_001361501 | AAGCAGATGAAGCCACCCTG  GTCTGCACGGGAATGGTGAT | 3940-3959  4564-45 | 625 |
| BDNF | NM_001316310 | GAAGAGCTGCTGGATGAGGAC  TTCAGTTGGCCTTTTGATACC | 517-537  848-828 | 332 |
| Gdnf | NM_001301357 | TCACCAGATAAACAAGCGG  TTTCATAGCCCAAACCCAA | 297-315  471-453 | 175 |
| IL-6 | NM_001314054 | TTCCATCCAGTTGCCTTCT  CTCATTTCCACGATTTCCC | 103-121  275-25 | 173 |
| TNF-α | NM_013693 | CCAGACCCTCACACTCAGA  GACAAGGTACAACCCATCG | 392-410  578-560 | 187 |
| IL-1β | NM_008361 | CCTGTGTCTTTCCCGTGGA  GGGTGTGCCGTCTTTCATT | 316-334  672-654 | 357 |
| IL-10 | NM_010548 | GCTCTTACTGACTGGCATGAG  CGCAGCTCTAGGAGCATGTG | 220-240  324-305 | 105 |
| ZO-1 | XM_036152895 | GGGAGGGTCAAATGAAGACA  GGCATTCCTGCTGGTTACAT | 6366-6385  6510-649 | 145 |
| Occludin | NM_001360538 | ATAATGGGAGTGAACCCGACG  CCACGATAATCATGAACCCCA | 724-744  910-890 | 187 |
| Claudin-1 | NM_016674 | TGGATGGCTGTCATTGGGG ACCTGGCATTGATGGGGGT | 562-580  688-670 | 127 |
| GAPDH | XM_036165840 | ACCACAGTCCATGCCATCAC  TCCACCACCCTGTTGCTGTA | 946-965  397-1378 | 452 |
| *Akkermansia muciniphila* | GCA_000020225.1 | CCTTGCGGTTGGCTTCAGAT | /  / | 329 |
|  |  | CAGCACGTGAAGGTGGGGAC |  |  |
